# Undergraduate students’ performance and perception of video-recordings versus live demonstrations for teaching orthodontic laboratory procedures: a randomized trial

**DOI:** 10.1186/s12909-025-07587-9

**Published:** 2025-07-04

**Authors:** Serene A. Badran, Iyad Al-omari, Abdelrahman Shqaidef, Zaid Al-Bitar, Ahmad M. Hamdan

**Affiliations:** 1 Department of Orthodontics, Pediatric and Community Dentistry, College of Dental Medicine, University of Sharjah, Sharjah, United Arab Emirates; 2 Department of Pediatric Dentistry and Orthodontics, University of Jordan, Amman, 11942 Jordan; 3 College of Dentistry, Center of Medical and Bio-allied Health Sciences Research, Ajman University, Ajman, United Arab Emirates; 4 Faculty of Dentistry, Al-Ahliyya Amman University, Amman, Jordan

**Keywords:** Dental education, Orthodontics, Wire bending, Practical skills, Video demonstration, Live demonstration

## Abstract

**Objectives:**

To compare three methods of delivering an orthodontic laboratory procedure on students’ academic performance; live demonstration, video-recorded demonstration, or both. To assess students’ perceptions and preferences to the demonstration methods employed.

**Materials and methods:**

A total of 202 fourth-year undergraduate students were randomly allocated to three groups; live demonstration, video-recorded demonstration, or both. Students were instructed to construct a buccal canine retractor immediately after the demonstration method ended, answer 2 short essay questions about buccal canine retractors to assess their theoretical comprehension, and fill a questionnaire to assess their perception and attitude towards the methods of demonstration.

**Results:**

The mean score for construction of the buccal canine retractor was significantly higher for the live demonstration group compared to the 2 other groups (*P* < 0.05). There were no significant differences between the 3 groups in answering the essay questions (*P* > 0.05). Most students (95.3%) agreed that live demonstrations helped them visualize and understand difficult wire bending techniques, and allowed interaction between students and the lecturer (93.9%). Around 83% agreed that video recordings were a useful aid to live demonstrations but only 11.9% indicated that they could totally replace live demonstrations. More than half of the students preferred a live demonstration compared to watching a video-recording.

**Conclusion:**

The live demonstration group performed better than the video-recorded demonstration group. Most students preferred a live demonstration method of teaching orthodontic wire bending, however the majority indicated that a video-recorded demonstration was a useful aid to a live demonstration.

## Introduction

Traditional live demonstration remains an essential teaching tool in dental education. However, there is a growing movement in healthcare education towards new approaches that help overcome the challenges facing education in the new century and the needs of the new generation of dental students [1]. One of these approaches include video-assisted practical instructions.

The benefits of a live demonstration include improved communication skills, increased students’ confidence [2] and better perception of practical procedures [3]. However, this method was found to have many drawbacks such as problems with visualization of the demonstration by the students, time limitations, the chance of missing critical steps and the non-repeatability of the demonstration [4, 5]. 

The video-assisted method has the advantages of providing better visualization at a closer distance [6], the ability of revising the recorded videos for future reference, and the advantage of overcoming shortage in faculty numbers [5]. 

While video-assisted teaching has been explored in several clinical dental specialties [2–4, 6–14], its benefits in teaching orthodontic manual skills has not been fully investigated. Two studies compared the effectiveness of both teaching methods on dental students’ orthodontic wire bending performances [5, 15] and concluded that both methods were equally effective. Sivarajan et al. [16] compared live demonstration to flipped classroom methods in teaching dental students wire-bending skills for six types of removable orthodontic appliance components. The flipped-classroom group scored higher on two of the six components (Adam’s clasp and Z-spring). However, two out of these three studies [5, 16] had a small sample size. A systematic review and meta-analysis comparing the efficacy between live and video demonstrations in undergraduate dental education has recently been published [17]. The authors aimed to assess the performance and satisfaction scores of undergraduate students in various dental disciplines. The studies they included showed mixed preferences for live and video demonstrations. Although the meta-analysis revealed that video demonstrations enhanced educational outcomes over live demonstrations, the high heterogeneity due to the variability in clinical procedures and sample sizes across studies limited meta-regression analysis of outcome variables. Accordingly, further randomized trials with high statistical power are recommended for robust conclusions [17].

A gap remains in the literature regarding the comparative impact of different teaching methods for orthodontic wire bending procedures on undergraduate dental students’ performance, perceptions, and preferences. Hence, the main aim of this study was to compare students’ performance using three methods of delivering an orthodontic laboratory procedure; live demonstration, video-recorded demonstration, or both. The second aim was to assess students’ perceptions and preferences to the different methods employed.

### Null hypothesis

There was no difference in laboratory performance between students attending a live demonstration, video-recorded demonstration, or both.

## Subjects and methods

This study was a multi-arm parallel group randomized trial with a 1:1:1 allocation ratio. There were no changes in the study design after the commencement of the study.

### Ethics approval and consent to participate

Ethical approval was obtained from the Academic Research Committee at the School of Dentistry - University of Jordan (reference number 75/2022/1419).

The targeted sample included fourth-year dental students enrolled in the Practical (laboratory) Orthodontics course at the School of Dentistry - University of Jordan, who had not previously failed the fourth year. An informed consent was obtained from the students who wished to participate.

Students were allocated into 3 groups: video-recorded demonstration group, who watched a video recording on the construction of a buccal canine retractor (BCR), using their mobile phones and a headset; live demonstration group, who watched the orthodontic technician construct the buccal canine retractor; and the combined demonstration group, who watched the video-recorded demonstration first then the live demonstration. The study took place in the orthodontic laboratory at the School of Dentistry.

### Sample size calculation

Sample size calculation was carried out using the G*Power computer software (G*Power, version 3.1.9.7; Heinrich-Heine University). A priori power analysis utilizing F test for one-way analysis of variance determined a total sample size of 180 students to obtain an effect size of 0.25, statistical power (1– β) of 0.85, and significance level (α) of 0.05. More students were recruited to compensate for any potential dropouts or exclusions.

### Randomization

Randomization was achieved first by dividing the students according to their performance (following university regulations) in orthodontic laboratory in the first semester into 3 groups; Very good (greater than 75), Good (from 66 to 75), and Satisfactory (less than 66). The randomization was then accomplished with a computer-generated list of random numbers. Random allocation to one of the three demonstration groups was carried out using a permuted random block size of 6. The allocation sequence was concealed in sequentially numbered, opaque, sealed envelopes for each laboratory group before the intervention. Laboratory group number was written on the outside of the envelops before opening them. The laboratory supervisor was responsible for opening the next envelop in sequence and implementing the randomization process.

### Blinding

One of the authors (IA) marked the constructed BCR blindly following standard set criteria for marking (Table 1). Another author (SB) wrote the questions and included a standard answer sheet to mark the scores accordingly. Answer sheets were corrected blindly. The orthodontic technician did not know the questions to avoid the possibility of giving hints to students during the demonstration. Blinding of students to the teaching method was not possible.

**Table 1:** Buccal canine retractor assessment criteria

1	Labial components are 1 mm away from buccal alveolar mucosa
2	Coil is at least 2 mm away from sulcus
3	Coil diameter 2–3 mm
4	There is no gap within the helix coil
5	Coil positioned distal to and above the active arm
6	Active arm should run parallel to the long axis of the canine to the middle of the crown
7	Horizontal retraction end of the mesial/active arm starts perpendicular to the mesial/active arm
8	Horizontal retraction end of the mesial/active arm curves along crown curvature to the level of mesial contact point of canine
9	The distal arm is carried through to the baseplate, in contact with the second premolar and above its contact area
10	0.5 to 1 mm clearance between retentive arm and palate, and retention tag facing towards the palate

### Intervention

The video-recorded demonstration was performed by an orthodontic laboratory technician and the video was recorded by a photographer and sound technician using a Sony HDR-XR520 240GB High definition Handycam Camcorder. The technician constructed the BCR in the same manner that he would during a live demonstration using an orthodontic manual, prepared by the instructors of the course, as a guide for the steps of construction to ensure consistency of the information presented. The educational video consisted of step-by-step instructions on constructing a buccal canine retractor, with close-up shots and voice-over explanations to enhance understanding.

Using the orthodontic manual as a reference, one of the researchers (SB) set two short essay questions for the students. Open-ended questions were used to avoid any possibility of students guessing the correct answer. In addition, students were asked to complete a questionnaire assessing their attitudes towards each learning method. A 5-point Likert scale was used to collect their answer which included; strongly agree, agree, neither agree or disagree, disagree, and strongly disagree. Students were asked to answer the questionnaire on a bubble sheet which was corrected using a bubble sheet scanner.

Fourth-year dental students must enroll in Practical (Laboratory) Orthodontics course and are usually divided into groups of 34–36 students; half of each group attends the laboratory session every other week. The duration of each session is 2 h. The study was conducted on 7 groups over a one-week period. All students in the 7 groups were invited to participate in the study during that week. In total, 205 students were enrolled in the study. The students were divided into three groups receiving different teaching methods. Allocation of students to each group was announced on the day of the study.

1. Live demonstration group:

Students attended the regular live demonstration, which lasted for 10 min. At the end of the demonstration, students were allowed to ask questions and then they were asked to construct the BCR within one hour and then answer the two essay questions and fill the questionnaire.

2. Video-recorded demonstration group:

The link to the video-recorded demonstration was sent to students’ mobile phones once they entered the orthodontic laboratory. Before watching the demonstration, students were given a headset (Beats EP On-Ear Headphones). The duration of the recording was 10 min. Students were allowed to stop and rewind the video if they wished. Once they had finished, they were asked to construct the BCR within one hour, and then answer the two essay questions and fill the questionnaire.

3. Combined demonstration group:

The video-recorded demonstration was sent to students’ mobile phones once they entered the orthodontic laboratory. Before watching the demonstration, students were given a headset (Beats EP On-Ear Headphones). The duration of the recording was 10 min. Students were allowed to stop and rewind the video if they wished. Then, students attended the regular live demonstration, which lasted for 10 min. Once they had finished, they were asked to construct the BCR within one hour, and then answer the two essay questions and fill the questionnaire.

Students in all three groups were given the same amount of time to construct the BCR and answer the essay questions.

### Outcome measures and any changes after trial commencement

The primary outcome measure for this study was student test scores (constructed buccal canine retractor score and two essay questions score) for the three groups of teaching methods.

The secondary outcome measure was students’ perception and preference towards traditional live demonstration and video-recorded demonstration methods.

### Statistical analysis

Differences between 3 study groups according to gender, academic achievement, and mean scores recorded for construction of a BCR and essay questions were studied using a One-Way ANOVA. Differences between individual groups were studied using a paired-samples T-test and One-Way ANOVA with least square difference (LSD) post hoc comparisons. The significance level for all statistical analyses was set at the 5% level (*P* < 0.05).

## Results

### Participant flow

**Fig. 1:**
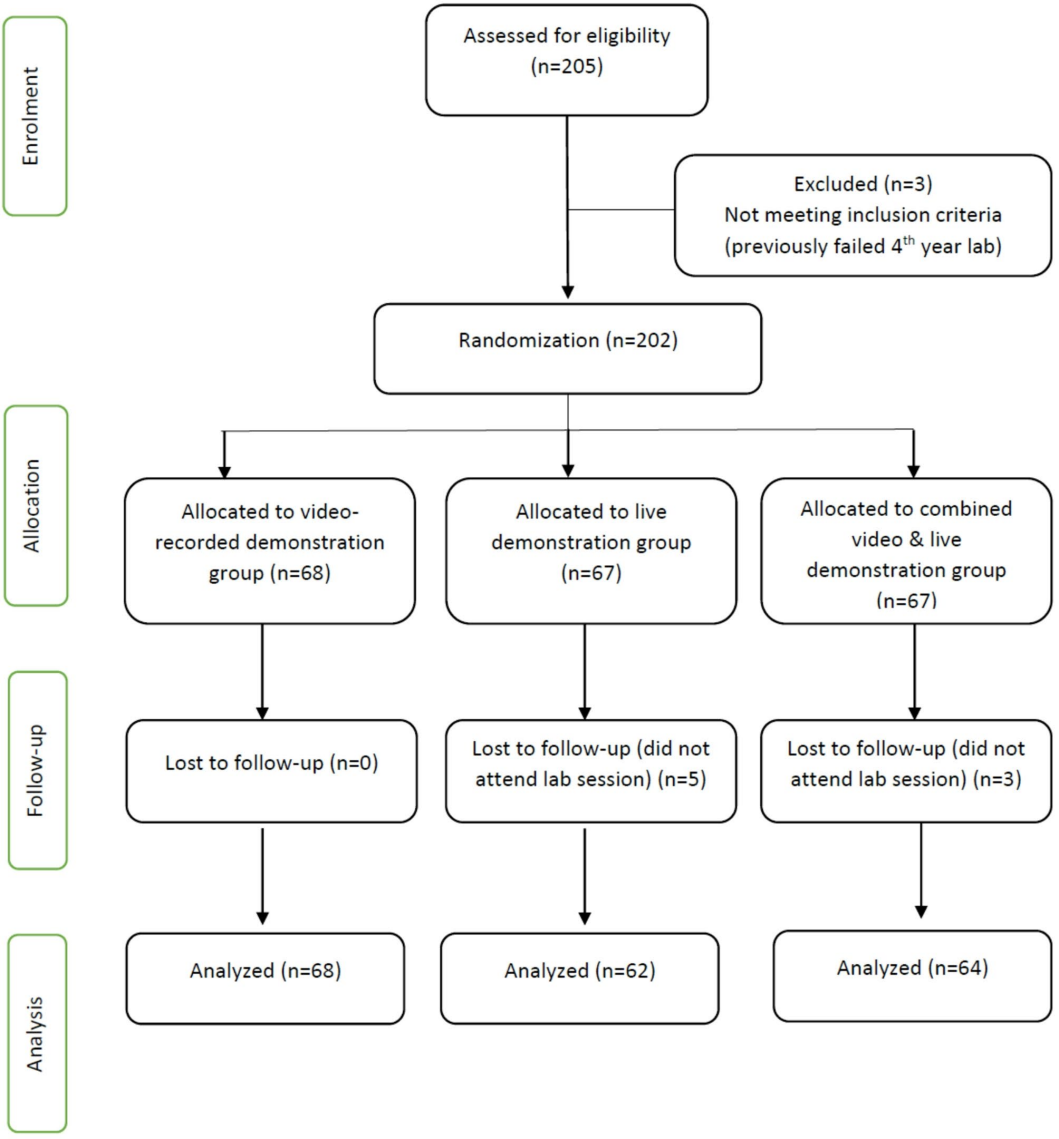
CONSORT diagram showing flow of participants in the study

Out of the 205 students enrolled in 4th year orthodontic laboratory course, 202 students met the eligibility criteria and were randomly allocated to one of the three groups. Five students from the live demonstration group and 3 students from the combined group did not attend the lab on the day of the study. In total, data from 194 students were included in the analysis stage of the study. Out of the 194 students, 68 belonged to the video-recorded group, 62 the live demonstration group, and 64 the video-live combined demonstration group (Fig. 1).

### Baseline data

Table 2 illustrates the distribution of students among the 3 study groups according to gender and academic performance. A One Way ANOVA showed no statistically significant differences among study groups for gender and academic performance (*P* > 0.05).

**Table 2:** Distribution of students according to gender and academic performance

Study Group	Female	Male	Total	Very Good	Good	Satisfactory
Video demonstration	53	15	68	25	26	17
Live Demonstration	44	18	62	16	27	19
Combined Live and video demonstration	44	20	64	18	24	22
Total	141	53	194	65	78	51

Table 3 shows the mean and standard deviation scores for assessments of dental students’ manual dexterity at constructing a BCR and answering 2 essay questions about BCRs. A One Way ANOVA showed no significant differences between group scores for answering the essay questions (*P* > 0.05). On the other hand, there were significant differences between study groups for scores of construction of the BCR and total scores (*P* < 0.05). Further comparison between each two groups revealed a significant difference between the live demonstration and the video recorded demonstration groups (*P* = 0.004) and the live and combined live-video demonstrations groups (*P* = 0.002); students who received a live demonstration performed better in the construction of the BCR than did the students in the other 2 groups (Table 4).

**Table 3:** Mean scores (standard deviations) for construction of a buccal canine Retractor and for the essay questions in each study group

Study Group	Construction of buccal canine retractor (Maximum of 10 points)	Essay questions (Maximum of 5 points)	Total score (Maximum 15 points)
Video recorded demonstration	5.7 (1.53)	3.9 (0.89)	9.4 (2.13)
Live demonstration	6.5 (1.37)*	4.0 (0.75)	10.3 (1.68)*
Combined Live and video recorded demonstration	5.6 (1.45)	3.7 (0.80)	9.4 (1.56)
Mean	5.9 (1.49)	3.9 (0.82)	9.7 (1.86)

*P < 0.05

**Table 4:** Comparison of students’ practical skills (constructed BCR scores) and theoretical knowledge (quiz scores) between study groups

	Practical Skills (Constructed BCR Scores)			Theoretical Knowledge (Quiz Scores)		
Compared groups	Mean Difference	Std. Error	*P*-value	Mean Difference	Std. Error	*P*-value
Video and Live demonstration groups	− 0.763	0.258	0.004*	− 0.1099	0.1463	0.453
Video and Combined live-video demonstration groups	0.071	0.255	0.780	0.1544	0.1432	0.283
Live and Combined live-video demonstration groups	0.835	0.260	0.002*	0.2643	0.1479	0.076

* *P* < 0.05

Table 5 illustrates the perceptions and preferences of the total sample of 4th-year dental students towards traditional live and video-recorded demonstrations. Most dental students (95.3%) agreed that live demonstrations helped them visualize and understand difficult wire bending techniques and that live demonstrations allowed interaction between students and the lecturer (93.9%). Conversely, around half the students (47.4%) indicated that it was difficult to see all the steps of a wire bending exercise during a live demonstration.

The majority of students (78.8%) did not think that live demonstrations were a waste of time, however, a similar proportion (78.3%) indicated that a video-recorded demonstration was useful because they could rewind, pause or play back the demo when needed, and half the students (55.7%) reported that a video-recorded demonstration allowed a clear view of all the steps of the wire bending exercise. Conversely, less than a quarter of students (21.2%) preferred watching a video recording compared to a live demonstration and only 11.9% of students indicated that video recording could totally replace live demonstrations. However, 83.5% of students reported that video recordings were a useful aid to live demonstrations (Table 5).

Finally, only 30% of students preferred learning wire bending from a video recording compared to a live demonstration, while 40.9% felt that a video recording was “worse” or “much worse” than a live demonstration teaching method, and 29% indicated that both methods of learning were “similar” (Table 5).

The students’ answers to the questionnaire were not significantly different between the three study groups, except for questions 7, and 10 (*P* < 0.05). Further comparison of students’ perceptions between each two groups confirmed these results except that answers to question 4 were significantly different between the video and the live demonstration groups (Table 6). More students in the live demonstration group did not agree that attending a live demonstration of a wire bending exercise is a waste of time (*P* < 0.05); while more of those who watched video demonstrations think that video demonstrations were superior to live demonstrations (Table 6).

**Table 5:** Comparison of dental students’ perceptions towards traditional live and video-recorded demonstration methods between the three study groups

Questions	^$^Answers (Frequency (%))					*P*-value
	1	2	3	4	5	
Q1. Live demonstrations help me visualize and understand difficult wire bending techniques						0.140
Video	40 (58.8)	22 (32.4)	6 (8.8)	0 (0)	0 (0)	
Live	45 (72.6)	16 (25.8)	1 (1.6)	0 (0)	0 (0)	
Video-Live	40 (62.5)	22 (34.4)	0 (0)	2 (3.1)	0 (0)	
Total	125 (64.4)	60 (30.9)	7 (3.6)	2 (1)	0 (0)	
Q2. Live demonstrations allow interaction between students and the instructor						0.409
Video	47 (69.1)	14 (20.6)	6 (8.8)	1 (1.5)	0 (0)	
Live	46 (74.2)	14 (22.6)	1 (1.6)	1 (1.6)	0 (0)	
Video-Live	39 (60.9)	22 (34.4)	2 (3.1)	1 (1.6)	0 (0)	
Total	132 (68.0)	50 (25.9)	9 (4.6)	3 (1.5)	0 (0)	
Q3. It is difficult to see all the steps of a wire bending exercise during a live demonstration						0.650
Video	15 (22.1)	23 (33.8)	10 (14.7)	15 (22.1)	5 (7.4)	
Live	8 (12.9)	18 (29.0)	21 (33.9)	10 (16.1)	5 (8.1)	
Video-Live	8 (12.5)	20 (31.3)	22 (34.4)	12 (18.8)	2 (3.1)	
Total	31 (16.0)	61 (31.4)	53 (27.3)	37 (19.1)	12 (6.2)	
Q4. Attending a live demonstration of a wire bending exercise is a waste of time						0.091
Video	4 (5.9)	2 (2.9)	12 (17.6)	25 (36.8)	25 (36.8)	
Live	0 (0)	1 (1.6)	7 (11.3)	25 (40.3)	29 (46.8)	
Video-Live	2 (3.1)	4 (6.3)	9 (14.1)	23 (35.9)	26 (40.6)	
Total	6 (3.1)	7 (3.6)	28 (14.4)	73 (37.6)	80 (41.2)	
Q5. Attending a video-recorded demonstration is useful because I can rewind, pause or play back the demo whenever I need to						0.860
Video	32 (47.1)	20 (29.4)	11 (16.2)	2 (2.9)	3 (4.4)	
Live	21 (33.9)	27 (43.5)	11 (17.7)	2 (3.2)	1 (1.6)	
Video-Live	27 (42.2)	25 (39.1)	7 (10.9)	4 (6.3)	1 (1.6)	
Total	80 (41.2)	72 (37.1)	29 (14.9)	8 (4.1)	5 (2.6)	
Q6. A video-recorded demonstration allows a clear view of all the steps of the wire bending exercise						0.682
Video	20 (29.4)	20 (29.4)	11 (16.2)	12 (17.6)	5 (7.4)	
Live	11 (17.7)	19 (30.6)	17 (27.4)	12 (19.4)	3 (4.8)	
Video-Live	14 (21.9)	24 (37.5)	11 (17.2)	9 (14.1)	6 (9.4)	
Total	45 (23.2)	63 (32.5)	39 (20.1)	33 (17)	14 (7.2)	
Q7. I prefer watching a video recording of a wire bending exercise compared to a live demonstration						0.026*
Video	12 (17.6)	11 (16.2)	15 (22.1)	17 (25.0)	13 (19.1)	
Live	0 (0)	8 (12.9)	19 (30.6)	22 (35.5)	13 (21.0)	
Video-Live	5 (7.8)	5 (7.8)	17 (26.6)	24 (37.5)	13 (20.3)	
Total	17 (8.8)	24 (12.4)	51 (26.3)	63 (32.5)	39 (20.1)	
Q8. A video recording of a wire bending exercise can totally replace live demonstrations						0.600
Video	4 (5.9)	5 (7.4)	15 (22.1)	20 (29.4)	23 (33.8)	
Live	0 (0)	5 (8.1)	10 (16.1)	28 (45.2)	19 (30.6)	
Video-Live	3 (4.7)	6 (9.4)	9 (14.1)	23 (35.9)	23 (35.9)	
Total	7 (3.6)	16 (8.3)	34 (17.6)	71 (36.8)	65 (33.7)	
Q9. A video recording of a wire bending exercise is a useful aid to a live demonstration						0.863
Video	30 (44.1)	22 (32.4)	9 (13.2)	6 (8.8)	1 (1.5)	
Live	21 (33.9)	34 (54.8)	4 (6.5)	3 (4.8)	0 (0)	
Video-Live	26 (40.6)	29 (45.3)	4 (6.3)	2 (3.1)	3 (4.7)	
Total	77 (39.7)	85 (43.8)	17 (8.8)	11 (5.7)	4 (2.1)	
Q10. How do you rate learning wire bending from a video recording compared to a live demonstration?						0.038*
Video	6 (8.8)	22 (32.4)	16 (23.5)	21 (30.9)	3 (4.4)	
Live	1 (1.6)	10 (16.1)	20 (32.3)	27 (43.5)	3 (4.8)	
Video-Live	5 (7.8)	14 (21.9)	20 (31.3)	23 (35.9)	2 (3.1)	
Total	12 (6.2)	46 (23.8)	56 (29)	71 (36.8)	8 (4.1)	

^$^ Answers for Questions 1 to 9 are: 1 = Strongly agree, 2 = Agree, 3 = Uncertain, 4 = Disagree, 5 = Strongly disagree. Answers to Question 10 are: 1 = Much better, 2 = Better, 3 = Similar, 4 = Worse, 5 = Much worse. *P < 0.05

**Table 6:** Comparison of students’ perception of demonstration methods between the study groups

Questions	Compared groups								
	Video and Live Demo			Video and Combined Demo			Live and Combined Demo		
	Mean Diff.	Std. Error	*P*-value	Mean Diff.	Std. Error	*P*-value	Mean Diff.	Std. Error	*P*-value
Q1	0.210	0.107	0.052	0.063	0.107	0.558	− 0.147	0.109	0.179
Q2	0.120	0.115	0.297	− 0.027	0.114	0.815	− 0.147	0.117	0.210
Q3	− 0.186	0.201	0.355	− 0.099	0.199	0.618	0.087	0.203	0.671
Q4	− 0.367	0.172	0.034*	− 0.091	0.171	0.594	0.276	0.175	0.116
Q5	− 0.069	0.172	0.688	0.023	0.171	0.893	0.092	0.175	0.598
Q6	− 0.188	0.215	0.384	− 0.074	0.214	0.728	0.113	0.219	0.604
Q7	− 0.528	0.207	0.012*	− 0.429	0.205	0.038*	0.098	0.210	0.640
Q8	− 0.193	0.191	0.313	− 0.100	0.189	0.599	0.093	0.193	0.629
Q9	0.089	0.166	0.591	0.052	0.164	0.750	− 0.037	0.168	0.827
Q10	− 0.447	0.176	0.012*	− 0.150	0.174	0.389	0.297	0.178	0.097

* *P* < 0.05

## Discussion

This study was conducted with the aim of finding the most efficient method of teaching a laboratory orthodontic course to undergraduate students. The design of the study avoids selection bias. Students were blinded to the method of teaching they were going to receive. Furthermore, clinicians were blinded when assessing the buccal canine retractor and the answers of the essay test.

The theoretical knowledge of students was assessed by means of two short essay questions. The use of essay questions as opposed to multiple choice questions reduced the possibility of guessing the correct answer. One author wrote and corrected the exam questions and was blinded to the method of demonstration that each student received, thus reducing any possible bias.

Students’ theoretical knowledge performance was comparable across the three groups. These findings were similar to some studies which compared video-recorded and live lectures [18, 19], although these studies compared didactic lectures and not practical courses. The few studies which compared different methods in teaching orthodontic wire bending [5, 15, 16] did not assess theoretical knowledge. Gorucu-Coskuner et al., [20] however, conducted a study to compare the effectiveness of live-video and video demonstration methods in training dental students in orthodontic emergency applications. They concluded that most students strongly agreed that both live-video and video demonstration enhanced their clinical knowledge.

In contrast, when investigating the practical performance of students in our study, the live demonstration group scored higher than the video and live-video demonstration groups. This may indicate that a live demonstration keeps students more attentive knowing that they have no other means of re-viewing the demonstration. This finding was in agreement with the study of Ramlogan et al. [6] but in disagreement with other studies [5, 13, 15]. Thilakumara et al. [13] compared video to live demonstration in teaching a prosthodontic laboratory course where third-year dental students were asked to arrange artificial teeth and found no significant differences between the two groups. Similarly, Alqahtani et al. [5] compared video to live demonstration in teaching an orthodontic laboratory course were students were asked to fabricate an Adam’s clasp and found no significant differences between the two groups. Atik et al. [15] compared a live-video to a conventional live demonstration in teaching dental students how to bend an orthodontic vestibular arch and found no significant differences in the performance of the 2 groups. The differences between the findings of the above-mentioned studies and ours may be attributed to the previously acquired skills between groups. Our study groups were matched for their manual performance skills and had been trained in constructing wire components (other than the BCR), while the aforementioned studies did not stratify their sample according to performance of the students. This may have introduced bias in their study. Moreover, their students had never done any wire bending, while our students had some experience in bending wires.

One of the advantages of a video-recorded demonstration is that the recorded video can be paused or watched again; this was confirmed by over 78% of our students in this study and corroborated by several other studies [1, 10, 15, 21]. 

Despite these benefits, live demonstrations give students that opportunity to interact with the instructor, which may enhance understanding of the wire-bending procedure; [15] that was found to be the reason for preferring live demonstrations in some studies [4, 9, 15]. Other studies found that live demonstrations improved communication skills [2, 22]. The majority of our students agreed that live demonstrations helped them visualize and understand difficult wire bending techniques which is in concurrence with the findings of Packer et al. [2] They also agreed that live demonstrations allowed them to interact with the instructor. A disadvantage of live demonstration reported by around half of the students in the current study is that it is difficult to see all the steps of a wire bending exercise during a live demonstration. This is in agreement with Aragon and Zibrowski [10] in which students in the clinic, who were given a copy of a video, reported that they could see the demonstration better on video compared to a live demonstration with students crowded around the demonstrator.

Less than a quarter of students preferred watching a video recording compared to a live demonstration and only 11.9% of students indicated that video recording could totally replace live demonstrations. The actual performance of students in this study does reflect their attitude towards video recording; the live demonstration group scored higher on the construction of the BCR than did the video demonstration group, while 41% of the students rated learning wire bending from a video-recording as “worse” or “much worse” compared to a live demonstration. Nonetheless, 83.5% of students reported that video recordings were a useful aid to live demonstrations. This illustrates that students support video-recorded demonstrations as an aid, but not as a replacement, to live demonstrations. Our findings were supported by other studies [5, 7, 12]. Packer et al. [7]. found that students who watched a video-taped versus a live demonstration of clinical and laboratory technical stages for constructing a removable partial denture did not differ in their assessment and understanding. However, they preferred live demonstrations and gave them higher scores for usefulness in aiding understanding of all stages of the altered cast technique [7]. 

Our students’ perceptions to the method of teaching did not differ much between the study groups. There was a tendency, however, for students who watched a video demonstration to rate video demonstrations as superior to live demonstrations. A similar finding was reported in another study, where students considered videos to be a better teaching tool than live demonstrations [13]. Atik et al. [15]. found that live-video was as effective and helpful as live demonstration, but their live-video demonstration also included the instructor’s live participation, thus differing from the video method employed in the present and previous studies. Furthermore, around 65% of their students reported that the teaching method was easy and understandable in both groups [15]. 

Alqahtani et al. [5] found that students gave a higher positive response to question related to clarity and ease of understanding in the video group compared to the live demonstration group. Their findings were not confirmed by ours in this study where most students reported that live demonstrations helped them visualize and understand difficult wire bending techniques, and allowed interaction between students and the instructor. At the same time, most of our students agreed that attending a video-recorded demonstration is useful because they can rewind, pause or play back the video demonstration whenever they need to. They also reported that a video-recorded demonstration allows a clear view of all the steps of the wire bending exercise. These findings were corroborated by a recently published systematic review and meta-analysis, evaluating the impact of live and video demonstrations on undergraduate dental students’ performance and satisfaction [17]. They revealed that both methods have distinct advantages in educational settings; Video demonstrations were praised for their clarity and the ability to pause and review content, enhancing students’ understanding of clinical procedures, while live demonstrations were favored for their interactive nature, allowing real-time engagement and guidance from instructors [17]. Similar findings were reported by other studies [12, 14] where most of the students believed that videos alone were not as effective as face-to-face clinical teaching and that videos should not replace live teaching [12]. 

While a recent systematic review indicated that psychomotor skills knowledge were significantly improved following video demonstrations compared to live demonstrations, they were unable to draw robust conclusions due to high heterogeneity of the studies [17]. The limited studies, the variability in the clinical procedures and the questionnaires used to assess students’ satisfaction limited meta-analysis of outcome variables [17]. 

The limitations of the present study include that it was a one-time assessment on one orthodontic wire component. Moreover, theoretical assessment was done directly after the demonstration ended; long term retention of information was not assessed. Furthermore, this study was conducted in one university in Jordan which may limit its generalizability.

## Conclusions

- Performance of the live demonstration group in constructing an orthodontic removable appliance component (BCR) was better than the video-recorded demonstration group, however, they showed equal understanding of theoretical knowledge.
- While the vast majority of students commended live demonstrations for helping to visualize and understand difficult wire bending techniques, video recordings were praised for their clarity and the ability to pause and review content.
- Most students preferred live demonstrations for their interactive nature, while a significant number acknowledged the usefulness of video recordings as supplementary aids.

## Data Availability

All data generated or analysed during this study are included in this published article.

## Abbreviations

BCR: Buccal canine retractor
